# Targeting mucin hypersecretion in COVID-19 therapy

**DOI:** 10.4155/fmc-2021-0111

**Published:** 2022-03-22

**Authors:** Parteek Prasher, Mousmee Sharma

**Affiliations:** ^1^Department of Chemistry, UGC Sponsored Centre for Advanced Studies, Guru Nanak Dev University, Amritsar, 143005, India; ^2^Department of Chemistry, Uttaranchal University, Arcadia Grant, Dehradun, 248007, India; ^3^Department of Chemistry, University of Petroleum & Energy Studies, Energy Acres, Dehradun, 248007, India

**Keywords:** COVID-19, mucins, SARS-CoV-2

The primary symptoms pertaining to the SARS-CoV-2-mediated cytokine storm involve hypersecretion of mucins, which potentially triggers various inflammatory pathways, contributing toward COVID-19 pathogenesis. Mucins play a central role in the front-line defense of the respiratory tract in response to physiological stimuli and by serving as a primary site of contact against the invading microbes. Mucins contain antimicrobial lactoferrin, lysozyme and salivary agglutinin that offer antiviral properties under optimal physiological conditions to sustain homeostasis. The airway epithelia, consisting of an outer gel-forming mucin layer and an inner layer of membrane-anchored mucins, serve as a potential binding site for the attacking pathogens and may facilitate the membrane fusion and cellular transit of SARS-CoV-2 [1]. When the viral load is high, overproduction of mucins becomes problematic as it impedes the lumen of the respiratory tract and limits optimal airflow. Furthermore, the hypersecretion of mucins causes ciliary dysfunction that thwarts respiratory clearance, manifesting as chronic obstructive pulmonary disease, asthma and ciliary dyskinesia that further aggravate the COVID-19 symptoms [1]. The primary symptoms pertaining to the SARS-CoV-2-mediated cytokine storm involve hypersecretion of mucins, which potentially triggers various inflammatory pathways contributing toward COVID-19 pathogenesis [1]. The occurrence of mucins in the bronchoalveolar lavage fluid and lung epithelial cells of COVID-19 patients further supports their role in COVID-19 pathophysiology. The association of mucus hypersecretion with the pathophysiology of COVID-19 makes it an interesting target for preventing virus propagation and disease transmission, and for the effective management of COVID-19. The mucus lining serves as the first line of defense in respiratory diseases, and its hypersecretion serves as a front-line barrier that hinders the realization of an optimal therapeutic delivery to the airways during COVID-19 infection. It is apparent that the COVID-19 management paradigm necessitates the development of novel delivery systems that successfully penetrate the mucus barrier to reach the underlying morbid tissues for delivering an optimal healing effect. Similarly, therapeutic formulations with mucoadhesive properties prolong the drug release to the target site, which lowers the dosing frequency, maintains an optimal therapeutic concentration of the drug at the target site and hence improves patient compliance [1]. In this commentary, we emphasize targeting the hypersecretion of mucins for containing the COVID-19 pandemic.

The COVID-19 pandemic continues to generate soaring morbidity and mortality with the occurrence of a second wave, pertaining to the transmission of the infection through fomites, opening economic activities, emerging SARS-CoV-2 variants, incomplete vaccination and poor compliance with the preventive measures [2]. Large-scale vaccination programs are ongoing in several parts of the world; however, the behavior of the innate immune response to the entry of an inactive virus form in the host cells is still ambiguous [2]. Surprisingly, some giants in the vaccine industry, including Merck, dropped their vaccine development programs, while GlaxoSmithKline and Sanofi decided to reperform their early-stage trials after the identification of anomalies in the dose requirement [3]. Pfizer and BioNTech successfully developed COVID-19 vaccines, but the US FDA did not approve the one developed by AstraZeneca, questioning the results from a large-scale trial [3]. Remdesivir, originally developed for the treatment of hepatitis C and Ebola virus, is the only licensed repurposed antiviral drug aimed at relieving COVID-19 symptoms; however, a lack of robust evidence to validate its usability in COVID-19 led WHO to issue a conditional recommendation for its use in critically ill patients [3]. Furthermore, the rationally designed molecules targeting SARS-CoV-2 main protease, structural spike (S) protein, and viral spike glycoproteins are in process and should deliver clinical results in the coming months [4,5]. The ambiguity around a successful medication paradigm continued with an attempt to use nonsteroidal anti-inflammatory drugs for targeting inducible COX-2 in COVID-19 subjects, which proved inconsequential in delivering the desired outcomes [6].

Targeting of the heightened innate immune response following the cellular entry of SARS-CoV-2 has proved highly beneficial in achieving symptomatic relief in COVID-19 infection. Heavily glycosylated mucins secrete mucus in response to the entry of pathogen to the respiratory tract, following which the co-ordinated ciliary movements on the respiratory epithelia lead to the exit of the invading pathogen from the airway passages. During heavy viral load, the hypersecretion of mucus and mucins causes obstruction of the airways and may manifest as airway infections. Reportedly, COVID-19 infection triggers the allergic reaction in the respiratory tract mucosa, thereby activating the hypersecretion of mucins, which alters the chemical structure of the former and hence enables the entry of SARS-CoV-2 to the host cells. On entering the cells, SARS-CoV-2 recruits neutrophils, cytokines and chemokines and instigates the mucus-mediated proinflammatory cascades that cause a cytokine storm [7]. In one study a significant 33% of autopsies performed on COVID-19 patients indicated severe mucoid tracheitis, which indicates respiratory failure caused by mucin hypersecretions [7]. Importantly, the inflammatory response to the cellular entry of SARS-CoV-2 causes an overexpression of mucins through STAT-mediated signaling, MAPK-mediated signaling and NF-κB-mediated signaling [7].

The investigations performed by Lu *et al.* by optical coherence tomography via bronchoscopy on critically ill COVID-19 patients indicated heightened levels of the mucins MUC5AC, MUC1 and MUC1-CT; notably, MUC1 is a membrane-anchored mucin expressed on the apical surface of the airway epithelial cells, while MUC1-CT is expressed on the cytoplasmic side of these cells [8]. Therefore the elevated cytoplasmic fragments in the sputum of COVID-19 patients confirms the presence of detached and disrupted lung epithelia. Alveolar damage with fibromyxoid exudates and upregulated macrophage infiltration in the lung tissues of COVID-19 patients further confirm these observations [9]. The increased expression of MUC1 in the patients who succumbed to the COVID-19 infection further necessitates the expansion of clinical trials that involve the use of MUC1 inhibitors to treat patients with COVID-19 [9]. Liu *et al.* investigated the onset of the overexpressed mucin-induced silent hypoxia in SARS-CoV-2-infected mice and macaques. The SARS-CoV-2 infection led to the induction of IFN-β and IFN-γ which activated the IDO-Kyn pathway-dependent aryl hydrocarbon receptor signaling, eventually causing an upregulation of the transcriptional expression of both membrane-tethered and secreted mucins in the alveolar epithelia. Apparently, the build up of alveolar mucus adversely affected the blood–gas barrier, hence inducing hypoxia and diminished lung capacity [10].

Zhang *et al.* reported the mucin-type O-glycosylation landscapes of SARS-CoV-2 S proteins [11]. The heavily glycosylated S proteins exposed on the SARS-CoV-2 surface support the attachment, entry and fusion of the virus with host cell membranes. Site-specific O-glycosylation profiling of SARS-CoV-2 S proteins identified 43 non-sialylated O-glycosites in the insect cell-expressed S protein and 30 sialylated O-glycosites in the human S protein. The investigations revealed that the clustered mucin-type O-glycans modified the SARS-CoV-2 S protein, where the O-glycan and O-glycosite composition varied with the host cell type [11]. He *et al.* reported mucin-induced bronchoalveolar dysfunction in COVID-19 patients [12]. The SARS-CoV-2 virus caused a hypersecretion of mucins mediated through the innate immune regulators IL-1β and TNF-α in the club cells. These events resulted in the development of acute respiratory distress syndrome (ARDS) in the COVID-19 patients. Notably, ATP synthesis abruptly increased in the SARS-CoV-2-infected patients, thereby elevating the levels of extracellular ATP which thwarted the co-ordinated ciliary movement in the airways. Gene ontology analysis further validated these claims. Eventually, the diminished ciliary maintenance and increased ATP production in the COVID-19 patients caused a 1000-fold augmentation in the airway mucin secretion [12].

Alimova *et al.* performed a drug repurposing analysis on fostamatinib as a candidate drug for managing the mucin-induced acute lung injury and ARDS caused during the COVID-19 infection [13]. R406, an active metabolite of fostamatinib, reportedly reduces the MUC1 protein levels in the lung epithelia of patients diagnosed with an acute lung injury; it functions by the inhibition of spleen tyrosine kinase, necessary for the expression of several proinflammatory cytokines. The severe symptoms of COVID-19 express as virus-induced pneumonitis, followed by a sustained release of cytokines and macrophage-derived IL-6, which upregulates MUC1 in human colon cancer cell lines. These findings supported the IL-6-mediated upregulation of MUC1 in the lungs of SARS-CoV-2-infected patients. The investigations on the critically ill COVID-19 patients with ARDS and sepsis indicated a negligible statistically significant difference in the circulating levels of interleukins and TNF-α, suggesting that the COVID-19-associated ARDS occurs due to the heightened inflammatory cytokine levels. The serum concentrations of KL-6/MUC1 in the terminally ill COVID-19 patients validated KL-6/MUC1 as a prognostic biomarker of the severity of COVID-19 disease. The upregulation of KL-6/MUC1 during acute lung injury and ARDS led to the hypothesis that there may be beneficial effects of fostamatinib in patients suffering from COVID-19 lung injury. The authors obtained *in vitro* confirmation of the inhibition of spleen tyrosine kinase by fostamatinib which triggered the removal of MUC1 from the surface of lung epithelial cells in mouse models of acute lung injury, which further supported the clinical trials of this drug’s efficacy against COVID-19 lung injury [13].

Muller *et al.* studied the effect of the marine natural product PolyP on the human alveolar basal epithelia cell line A549 in the mucin environment [14]. The cell lines expressed mucins, ectoenzymes, alkaline phosphatase and adenylate kinase, involved in the extracellular generation of ATP from PolyP. Immunostaining analysis confirmed that the addition of PolyP to the collagen-based hydrogel increased the production of membrane-anchored MUC1 and the secreted mucin MUC5AC. The *in vitro* analysis established that PolyP could exert a protective effect against SARS-CoV-2 by stimulating the innate immune response, strengthening the mucin barrier with the antimicrobial proteins lactoferrin and lysozyme. Moreover, PolyP inhibited the attachment of virus to the host cells, as indicated by the diminished binding strength between the viral receptor-binding domain and cellular ACE2 receptor. Apparently, the increased level of mucins in response to the exposure to PolyP induced the secretion of lactoferrin and lysozyme from the airway epithelial cells. A surge in the accumulation of these antimicrobial proteins in the airway mucus functions to break the resistant viral envelope [14].

Furthermore, the mucin signature serves as a tool for the prediction of susceptibility to COVID-19, enabling differentiation of low- and high-risk patients. The glycome and mucin signature play a significant role in the determination of susceptibility, disease progression and response to therapy in the COVID-19 patients [15]. This enables a proactive approach in countering the imminent dangers posed by the successive waves of COVID-19.

## Conclusion

The emergence of successive waves of COVID-19 prompted the identification of novel approaches for effectively managing the further spread of the pandemic. Mucus hypersecretion during SARS-CoV-2 infection represents an intense immune response, which further proves detrimental to the well-being of the patients. Mainly, the hypersecreted mucus impedes drug delivery to the target infection site in the airways, as contemporary delivery vehicles fail to penetrate the thick mucus wall or become entrapped in the mucus mesh, followed by their sweeping away by the physiological mucociliary mechanism. Hence the mucus hypersecretion presents a major barrier in COVID-19 therapy and serves as a desirable target for the development of an effective COVID-19 management paradigm. Apparently, the mucus-penetrating or mucus-adhering particles provide an added advantage by maintaining an optimal drug concentration at the infected site in the airways, in addition to preventing virus transmission through the infected mucus released during coughing and sneezing. Targeting mucus hypersecretion during SARS-CoV-2 infection therefore provides an effective treatment strategy to improve COVID-19 mortality and morbidity, and to prevent the propagation of successive waves of the pandemic by preventing disease transmission.

## References

[1] Prasher P, Sharma M. Mucoadhesive nanoformulations and their potential for combating COVID-19. Nanomedicine 16(28), 2497–2501 (2021). 34730403 10.2217/nnm-2021-0287 PMC8577509

[2] Perumal AKK, Kharlukhi J, Omar UF. The second wave of COVID-19: time to think of strategic stockpiles. Can. J. Pub. Health 111, 486–487 (2020). 32705521 10.17269/s41997-020-00371-w PMC7376820

[3] Bar-Zeev N, Inglesby T. COVID-19 vaccines: early success and remaining challenges. Lancet 396(10255), 868–869 (2020). 32896290 10.1016/S0140-6736(20)31867-5 PMC7471800

[4] Gil C, Ginex T, Maestro I COVID-19: drug targets and potential treatments. J. Med. Chem. 63, 12359–12386 (2020). 32511912 10.1021/acs.jmedchem.0c00606

[5] Sharma M, Prasher P, Mehta M Probing 3CL protease: rationally designed chemical moieties for COVID-19. Drug. Dev. Res. 81, 911–918 (2020). 10.1002/ddr.21724 32729640

[6] Prasher P, Sharma M, Gunupuru R. Targeting cyclooxygenase enzyme for the adjuvant COVID-19 therapy. Drug Dev. Res. 82, 469–473 (2021). 33496060 10.1002/ddr.21794 PMC8013002

[7] Khan MA, Khan ZA, Charles M Cytokine storm and mucus hypersecretion in COVID-19: review of mechanisms. J. Inflamm. Res. 14, 175–189 (2021). 33519225 10.2147/JIR.S271292 PMC7838037

[8] Lu W, Liu X, Wang T Elevated MUC1 and MUC5AC mucin protein levels in airway mucus of critical ill COVID-19 patients. J. Med. Virol. 93, 582–584 (2021). 32776556 10.1002/jmv.26406 PMC7436726

[9] Jamaly S, Tsokos MG, Bhargava R Complement activation and increased expression of Syk, mucin-1 and CaMK4 in kidneys of patients with COVID-19. Clin. Immunol. 229, 108795 (2021). 34252574 10.1016/j.clim.2021.108795 PMC8270746

[10] Liu Y, Lv J, Liu J Mucus production stimulated by IFN-AhR signaling triggers hypoxia of COVID-19. Cell Res. 30, 1078–1087 (2020). 33159154 10.1038/s41422-020-00435-z PMC7646495

[11] Zhang Y, Zhao W, Mao Y O-glycosylation landscapes of SARS-CoV-2 spike proteins. Front. Chem. 9, 689521 (2021). 34552909 10.3389/fchem.2021.689521 PMC8450404

[12] He J, Cai S, Feng H Single-cell analysis reveals bronchoalveolar epithelial dysfunction in COVID-19 patients. Protein Cell 11(9), 680–687 (2020). 32671793 10.1007/s13238-020-00752-4 PMC7363016

[13] Alimova M, Sidhom EH, Satyam A A high content screen for mucin-1-reducing compounds identifies fostamatinib as a candidate for rapid repurposing for acute lung injury during the COVID-19 pandemic. Cell Rep. Med. 1(8), 100137 (2020). 33294858 10.1016/j.xcrm.2020.100137 PMC7691435

[14] Muller WEG, Neufurth M, Wang S, Tan R, Schroder HC, Wang X. Morphogenetic (mucin expression) as well as potential anti-corona viral activity of the marine secondary metabolite polyphosphate on A549 cells. Marine Drug 18(12), 639 (2020). 10.3390/md18120639 PMC7764923 33327522

[15] Bose M, Mitra B, Mukherjee P. Mucin signature as a tool to predict susceptibility to COVID-19. J. Mol. Biomark. Diagn. 11, 440 (2020). 10.14814/phy2.14701 PMC7771898 33373502

